# Ripen banana dataset: A comprehensive resource for carbide detection and ripening stage analysis to enhance food quality and agricultural efficiency

**DOI:** 10.1016/j.dib.2025.111659

**Published:** 2025-05-14

**Authors:** Elman Alam, Md Tarequl Islam, Ishrat Zahan Raka, Onamika Sarkar Ritu, Md Shakhawat Hossain, Wahidur Rahman, Rahat Khan

**Affiliations:** a Department of Computer Science and Engineering*,* Khwaja Yunus Ali University*,* Sirajganj*,* Bangladesh; b Department of Computer Science and Engineering*,* Mawlana Bhashani Science and Technology University*,* Tangail*,* Bangladesh; c Department of Computer Science and Engineering*,* Independent University of Bangladesh*,* Bangladesh; d Department of Computer Science and Engineering*,* Uttara University*,* Dhaka*,* Bangladesh

**Keywords:** Computer vision, Food safety, Image classification, Food quality assessment, Food ripening, Deep learning, Image analysis, Agricultural technology

## Abstract

We introduce the “Ripen Banana” dataset, a newly developed collection featuring two distinct classes of ripen banana images: carbide and non-carbide. The dataset contains images from raw to ripe bananas that have been ripened with carbide and without carbide. The images collected from various locations in Sirajganj, Bangladesh. It contains 1404 original images and 6410 augmented images, resulting in 7814 images overall. The original images were taken against carefully selected white backgrounds. This was made it by evaluating these images for their sharpness and brightness levels to ensure they meet the quality standards that are required. After that, we organize each image into specified subfolders to ensure simple and fast access for large range of machine learning and deep learning models. This dataset offers significant opportunities for advancements in agricultural practices, nutrition and the food chain, food environment monitor, and computer vision technologies. This “Ripen Banana”

dataset can be utilized to facilitate data-driven methods in food inspection, banana ripening systems, and post-harvest management. The dataset documents the stages of ripening, offering an opportunity to improve agricultural practices, improve the quality and safety of fruit production, and protect the food supply chain. The “Ripen Banana” dataset can be a valuable resource for future study in the fields of food science and nutrition, among people around machine learning background.

Specifications Table

**Table:** 

Subject	Computer Sciences
Specific subject area	Food Quality and Safety, Machine Learning Classification, Deep Learning Classification
Type of data	Raw, Analyzed, Filtered, Processed.
Data collection	To gather the dataset, we did a controlled experiment to track the ripening stages of bananas treated with calcium carbide compared to the ones which ripened under natural conditions. In August, the authors approached banana farmers from different regions of Sirajganj district in Bangladesh and collected bananas directly from their trees. The collected bananas were then divided into two groups: one group was professionally treated with calcium carbide, while the other group remained untouched. The study was performed between August 26 and September 2, 2024, using an iPhone 11 to consistently capture images under consistent lighting conditions. The study, which was finished within August 26 and September 2, 2024, used an iPhone 11 camera to capture images at 2-hour intervals in a white background environment. A total of 1640 images were taken, which produced 1404 high-quality images: 311 that contained carbide and 1093 without. Standard techniques including flipping, rotation, and brightness adjustment resulted in the collection of 7814 images, which can be used in machine learning applications relating to food quality and safety.
Data source location	We gathered this data from the cities nearby. For example: 1. Ullapara (6760), Sirajganj. 2. Sirajganj District (6700), Sirajganj. 3. Shahjadpur Upazila (6770), Sirajganj. 4. Belkuchi Municipality (6740), Sirajganj. 5. Enayetpur City (6751), Sirajganj
Data accessibility	Repository name: Mendeley Data [4] Data identification number: 10.17632/j9sp322drp.1 Direct URL to data: https://data.mendeley.com/datasets/j9sp322drp/1
Related research article	*None*

## 1 Value of the Data

• This dataset promotes machine learning applications related to food quality and safety by separating between naturally ripened and chemically ripened bananas.
• It allows development, validation, and analysis of classification models designed for detecting adulterants in fruits, solving an important issue with regard to food safety.
• It provides a carefully organized set of images that includes both original and enhanced data, which is useful for supervised learning in image classification

## 2 Background

In Bangladesh, some unscrupulous banana traders use calcium carbide to ripen bananas quickly, which are then sold in local markets. The use of calcium carbide causes contamination in the bananas, leading to various health issues for consumers, and in some cases even death. To address this problem, we have created “Ripen Banana” dataset that will identify banana contamination with carbide in the market. Another key objective of creating “Ripen Banana” dataset to offer an efficient, accurate and high quality labelled resource to distinguish banana ripen using calcium carbide and those ripen naturally resource which can help to develop of machine learning and deep learning solutions for agriculture and food science. This dataset has been created to provide useful information and it support the building of predictive models and algorithms that enhance the efficacy of bananas and meet the nutritional standard. The dataset contains two distinct classes of images of ripened bananas: the ones which was treated with carbide and those that was not. This dataset gives an appropriate structure, providing comprehensive information for the building of predictive algorithms and models that could significantly increase the understanding of banana ripening processes. The “Ripen Banana” dataset allows professionals to refine post-harvest management by applying data-driven strategies to optimize ripening techniques, which improve fruit quality and safety throughout the supply chain.

## 3 Data Description

This dataset on “Ripen Banana” describes the various stages of ripening in Musa sapientum, commonly known as the Sabri banana, looked at under two conditions: natural ripening and accelerated ripening using calcium carbide.

**Fig. 1:**
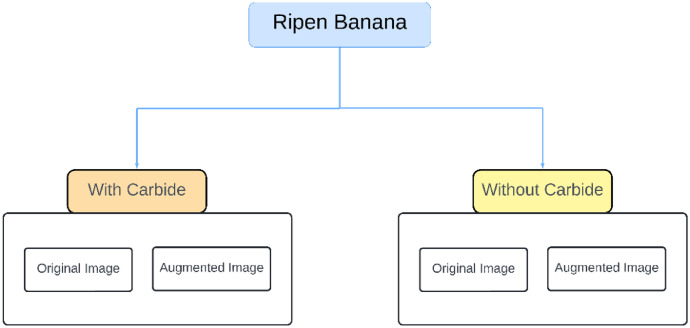
Proposed ‘Ripen Banana’ dataset directory layout.

**Fig. 2:**
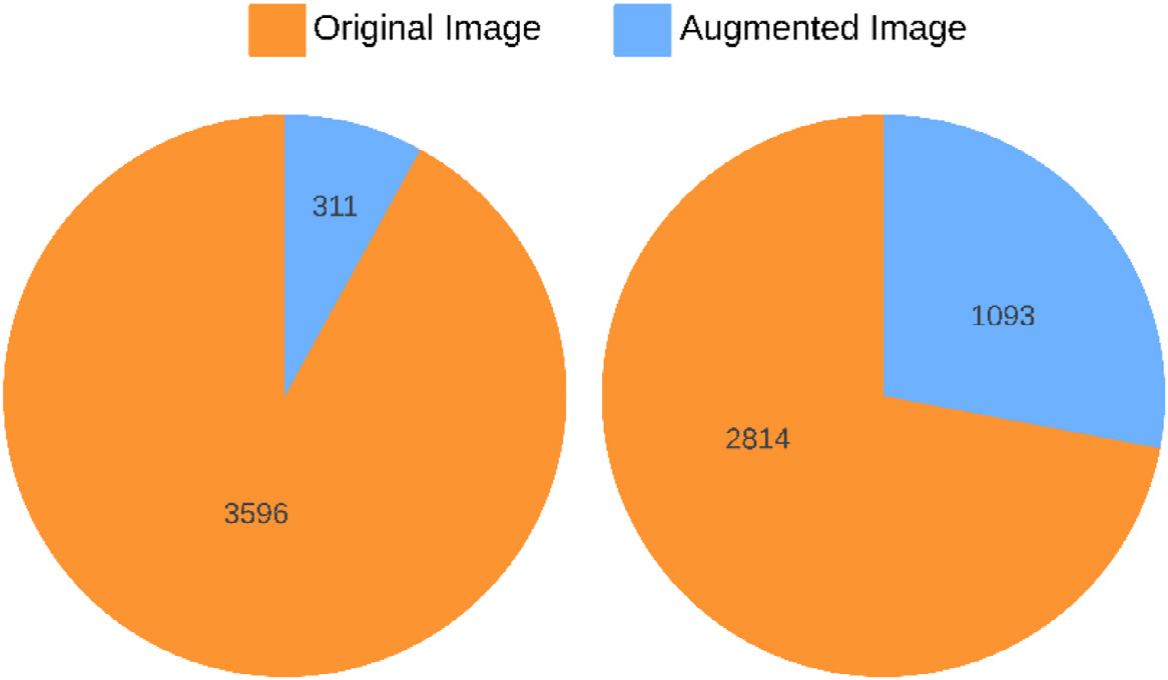
Distribution of original and augmented images in ‘*Rippen Banana’* dataset.

**Fig. 3:**
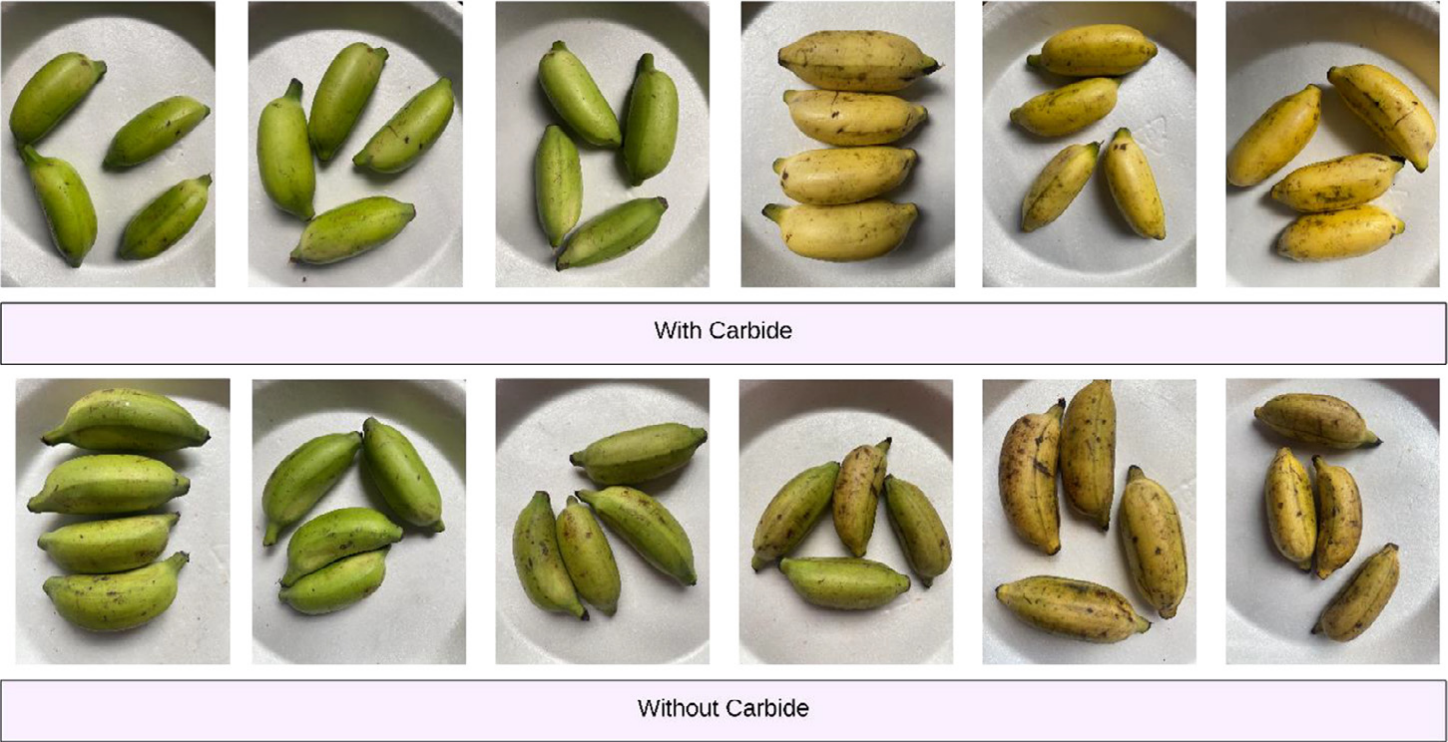
A representation images from every category of the ‘Ripen Banana’ dataset.

The dataset was gathered over a seven-day controlled experiment, starting on August 26, 2024, and ending on September 2, 2024. We captured 1404 original images using a phone camera at two-hour intervals after applying calcium carbide treatment and at selected intervals for naturally ripened bananas. The dataset includes 311 images of carbide-treated bananas that reached full ripeness by the second day, alongside 1093 images of naturally ripened bananas. Furthermore, techniques for data augmentation were utilized to achieve class balance, resulting in 3596 augmented images for the carbide-treated batch and 2814 augmented images for the naturally ripened batch shows Fig. 1, Fig. 2, Fig. 3 and Table 1.

**Table 1:** Summary of the Ripen Banana dataset.

Attribute	Description
Dataset Name	Ripen Banana Dataset
Banana Type	Musa sapientum (Sabri)
Ripening Conditions	Natural ripening and calcium carbide-induced ripening
Capture Interval	Every 2 h
Original Images	1404 total (311 with carbide, 1093 without carbide)
Augmented Images	6410 total (3596 with carbide, 2814 without carbide)
Background Setup	White background under natural diffused lighting
Geographic Locations	Ullapara, Sirajganj Sadar, Shahjadpur, Belkuchi, Enayetpur (Sirajganj, BD)
Annotation	Class-labeled folders (Carbide / Non-Carbide)
Usage Purpose	Machine learning & deep learning models for ripeness detection and food safety

With Calcium Carbide: Calcium carbide-ripened bananas ripen faster. People often use this method to guarantee that bananas arrive punctually and are ready for consumption. Calcium carbide promotes consistent ripening, while arsenic and phosphorus impurities pose health dangers. These green bananas turn golden and tender after treatment in a few days. Bananas with a creamy texture are delicious, but others say they taste better when they mature naturally and gain flavor and nutrients. Check labels and sources to ensure your bananas are safe and adequately ripened (Table 2).

**Table 2:** Accuracies for the pre-trained ResNet-50 and DenseNet-201 for *‘Ripen Banana’* dataset.

Model	Train Accuracy ( %)	Validation Accuracy ( %)	Test Accuracy ( %)
Pre-trained ResNet-50 (224 × 224 × 3)	72.98 %	72.40 %	72.36 %
Pre-trained DenseNet-201 (224 × 224 × 3)	79.26 %	78.75 %	80.04 %

**Fig. 4:**
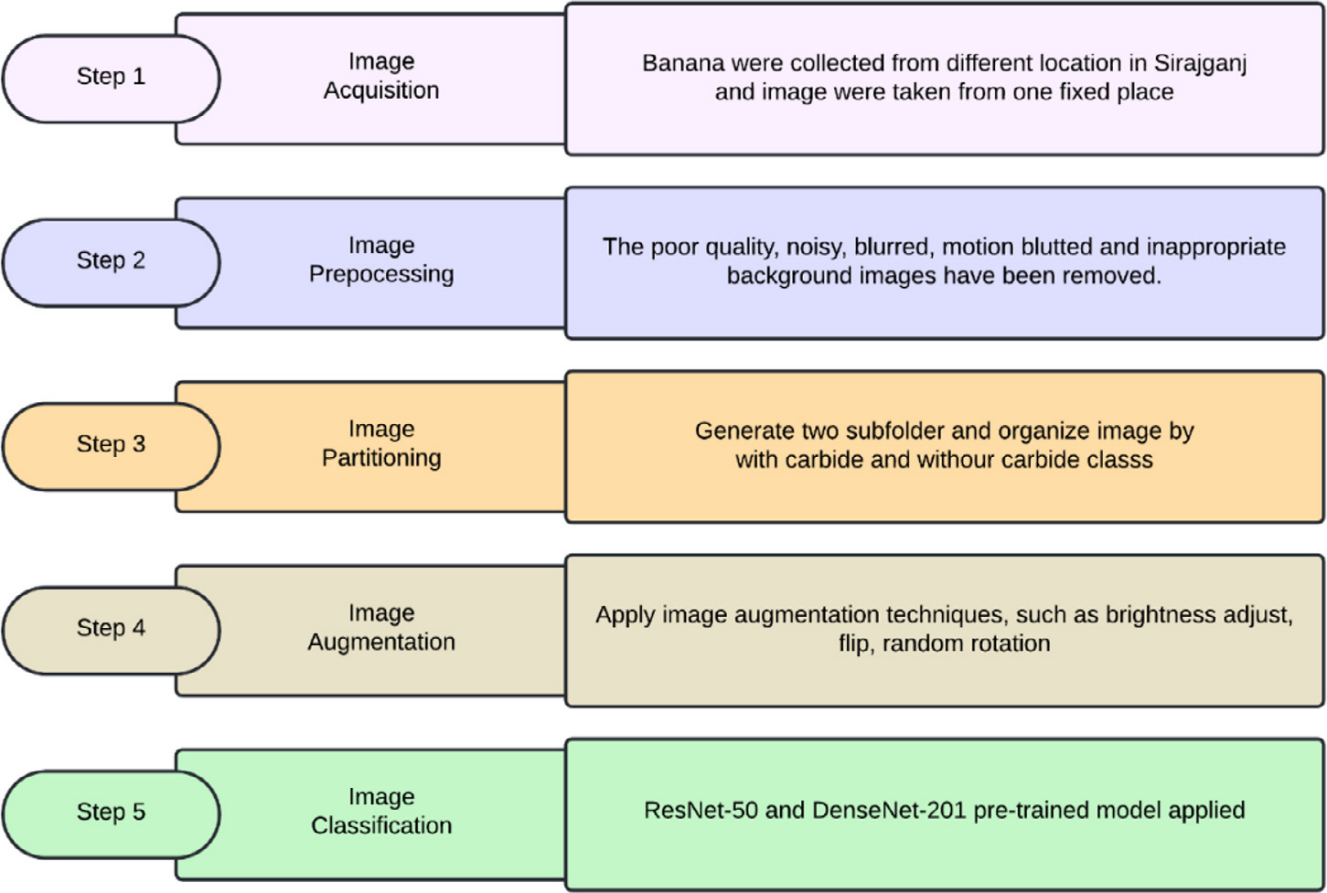
Processes involved in building the ‘*Ripen Banana’* dataset.

**Fig. 5:**
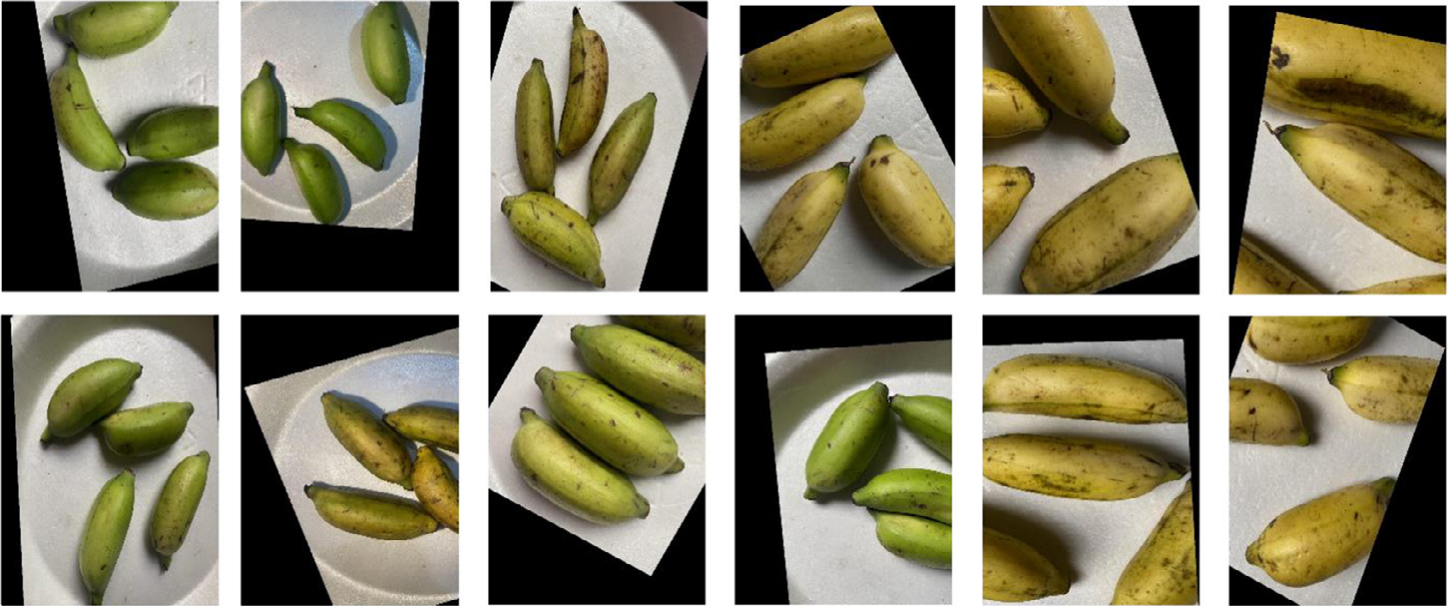
Several samples of augmented image.

**Fig. 6:**
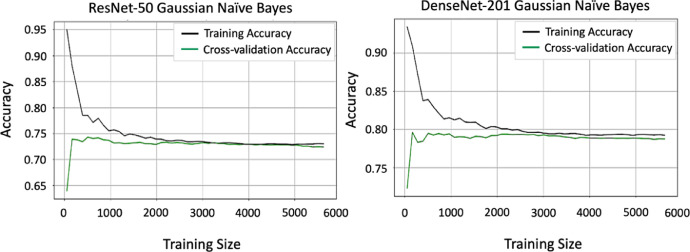
The accuracy of Resnet-50 and DenseNet-201 during training and validation.

**Fig. 7:**
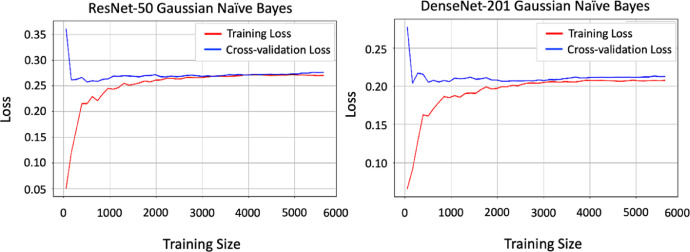
The loss for the ResNet-50 and DenseNet-201 models.

**Fig. 8:**
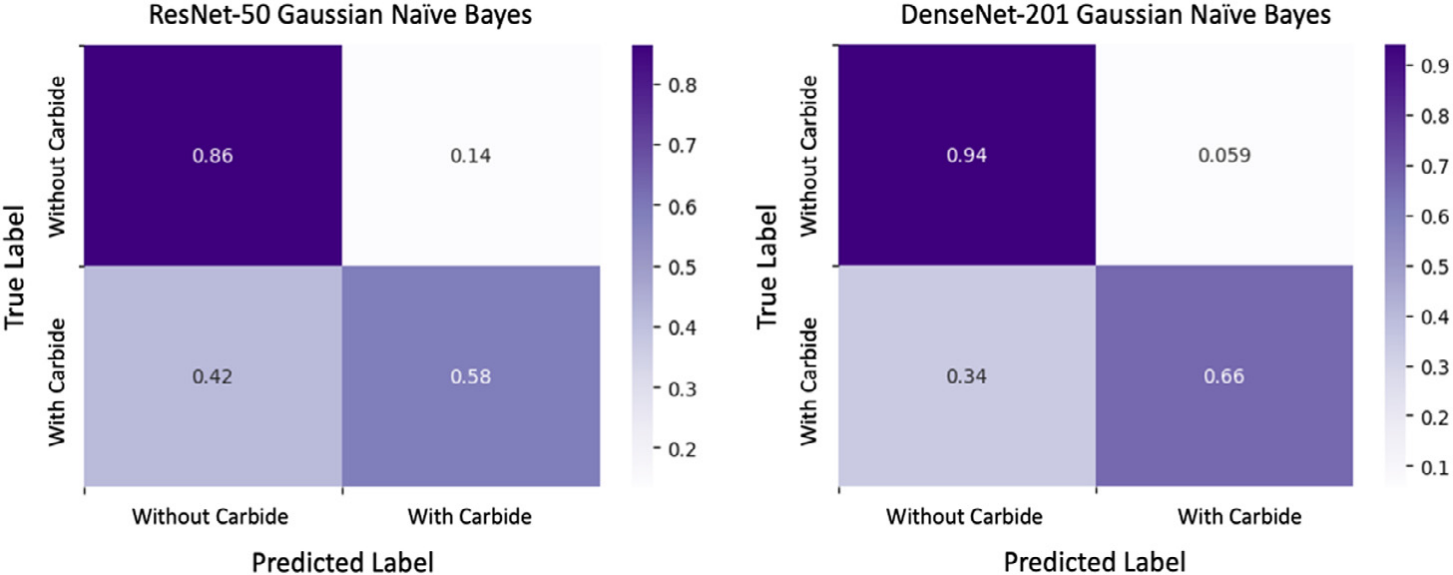
The confusion matrices for the ResNet-50 and DenseNet-201 models.

**Fig. 9:**
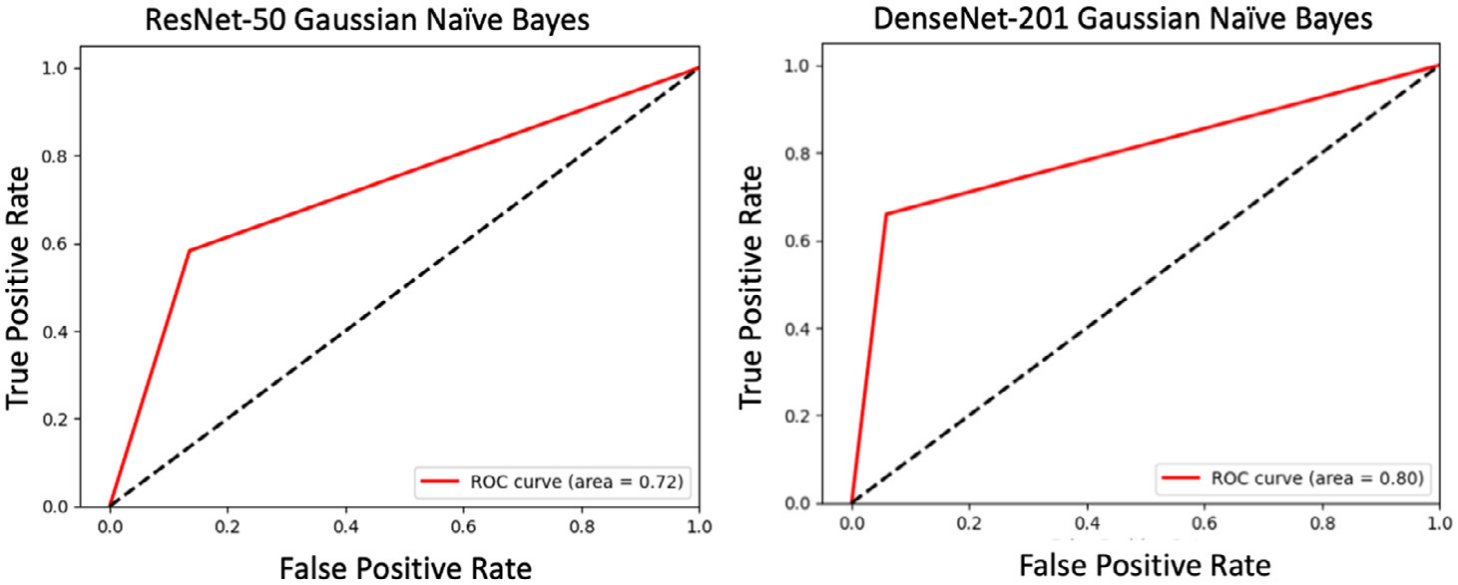
The receiver operating characteristic (ROC) curve for the ResNet-50 and DenseNet-201 models.

Without Calcium Carbide: Bananas that ripen naturally enhance their sweetness and flavor progressively through a natural ripening process. We harvested bananas while green and then allowed them to ripen at room temperature. During this process, starches transform into sugars, resulting in a softening of the fruit. A vibrant yellow hue with brown speckles emerges, signifying peak ripeness. People often view naturally ripening bananas as a healthier option due to their lack of chemicals, robust flavor, and creamier texture. Additionally, they usually possess a more attractive fragrance, enriching the overall consumption experience (Fig. 4, Fig. 5, Fig. 6, Fig. 7, Fig. 8, Fig. 9).

## 4 Experimental Design, Materials and Methods

Developing process of the “Ripen Banana” dataset involves five various phases: acquiring images, preprocessing images, partitioning images, augmenting images, and classifying images. This section offers a concise overview of every step.

### 4.1 Image acquisition

The dataset includes two distinct classes of ripe bananas. We gather the bananas from various locations in Sirajganj. The images of the raw ripened bananas were captured at the Khwaja Yunus Ali University Lab in Sirajganj District, Bangladesh, using an iPhone 11 equipped with a dual-camera system, including a 12 MP wide camera with an aperture of f/1.8 and a 12 MP ultra-wide camera with an aperture of f/2.4. We captured every image against a uniform backdrop of natural daylight. We employed diffused lighting throughout the image acquisition process to enhance the visibility of banana features and minimize shadow effects. We meticulously maintained a consistent and natural white background throughout the capture procedure Fig. 3. To keep the same standard regarding quality of image, we conduct consistent assessments all over the acquisition process. The process included confirming the point of focus, adapting the light source, and capturing images from various point of view to figure out deviations in bananas Fig. 4, Fig. 5, Fig. 6, Fig. 7, Fig. 8, Fig. 9.

### 4.2 Image preprocessing and quality evaluation

After taking the pictures, we carefully checked them and deleted any that had bad backgrounds or were missing parts of the bananas. Next, we reviewed the quality of the photos. The study of picture quality looked at sharpness and brightness. It specifically addressed problems like blurry focus, motion blur, and exposure control that occurred while taking the images. The sharpness and brightness of the images were checked by looking at the average width and height of edges, consistent with the method of analysis suggested by Hossain et al. [1]. This method has been used because of its reference-less nature, as recognizing an appropriate reference image for our study proved to be difficult. This important step made sure that only the best shots were chosen for more review. Removing these poor-quality pictures helped keep the dataset strong and made future studies more reliable. At first, we gathered 1630 pictures of two different kinds of ripe bananas. Afterwards, an extensive instruction review was carried out that removed images featuring inappropriate backgrounds or subjects. After that, we assessed the image quality through a computer program to exclude images that displayed unfit quality, including those with focus issues, motion blur, or exposure complications. Upon finalizing the image preprocessing and evaluation phase, our dataset comprises 1404 original images. Upon finalizing the image preprocessing and evaluation phase, our dataset comprises 1404 original images.

### 4.3 Image segmentation

After applying image preprocessing methods, the original collection comprises 1404 images. Subsequently, we established two separate directories of the banana's original image datasets. We then created two folders, one with carbide and the other without carbide. We systematically categorized and arranged the images into multiple folders based on whether they contained carbide. We randomly divided the original images into subsets for training, validation, and testing to train and assess the machine learning and deep learning models. The separation was executed at a ratio of 80:10:10 for every single classes.

### 4.4 Image augmentation

The performance and adaptability of models for classification as part of machine learning and deep learning algorithms has been improved by the use of many different kinds of image augmentation techniques. The dataset got improved by using the Keras ImageDataGenerator class to increase number of images. In order to make possible several kinds of augmentation techniques, including horizontal flipping, random rotation, and adding random noise, we set the fill_mode property to 'nearest'. We implemented image augmentation on all gathered images after organizing them into category-based folders for streamlined access.

### 4.5 Image classification

ResNet50 [2] and DenseNet-201 [3], two popular and lightweight Deep Learning (DL) models that are based on Convolutional Neural Network (CNN) architectures [4], were applied to determine the potential of the “Ripen Banana” dataset.

### 4.6 ResNet50 overview

The area of computer vision and deep learning were greatly impacted by ResNet50, an important set of convolutional neural network models. This layout is defined by its ability for collecting complex visual data, which is represented by 50 layers [5]. ResNet50′s development is unique by the application of a residual learning framework with quick connections which allow rapid transfer of information across the network. The direct flow solves the issue of disappearing, which is a common problem in deep neural networks, enabling ResNet50 to process complex designs more efficiently.

### 4.7 DenseNet-201 model overview

The DenseNet-201 model is a convolutional neural network architecture that densely connected uses parameters effectively to gain high performance [6]. As part of internal attention techniques, DenseNet-201 uses densely linked convolutional layers, which require that each layer is connected to every other layer. This helps the re-use of features and the effective gradient flow. This approach allows the model to acquire wide feature representations with only a small number of parameters.

DenseNet-201 is a well capable model for image classification roles, as it is capable of collecting difficult spatial and hierarchical data [7]. Given this, it is suitable for our dataset [8] classification task due to its robust design, which easily interacts with deep learning frameworks such as TensorFlow and PyTorch.

### 4.8 Dataset preparation

The models we trained using the “Ripen Banana” dataset it was split into three separate folders. The training set occupies 80 % of the data, the validation sets occupies 10 % and test sets occupies 10 %. Using the popular Python function Python_splitter, which effectively handles dataset splitting, helped manage this partition. Every subfolder in the train, validation, and test folders after partitioning has two subfolders representing our species classifications.

### 4.9 Data preprocessing

The Image Data Generator Python package pre-processes each image in the dataset before model training. The images are resized to (224 × 224) dimensions with three channels (RGB), as both ResNet-50 and DenseNet-201 are designed to fit an input size of 224 × 224 × 3.

### 4.10 Model training and evaluation

We initialized both models using pre-trained weights from ImageNet, freezing the top layers of both structures except the final dense layer. Using batch normalisation, we constructed two fully connected networks (FCNs) with 512 and 256 neurons, respectively, after extracting all the pre-trained features from both architectures using a global average pooling operation. We utilized the Rectified Linear Unit (ReLU) activation function in each dense layer and applied a dropout rate of 20 % to the neurons before the output layer to prevent overfitting. Our model's final, fully connected layer contains two neurons, one for each class in our dataset. We used convolutional and transformer-based architectures to capture different features in our dataset effectively Table 2.

## Limitations

The study has few limits. Sirajganj may not represent an accurate summary of the entire banana species or ripening conditions that exist in other regions. Risks arise regarding the use of calcium carbide for ripening banana, especially its amount and impact, which might affect the ripening process of fruits quality and safety. The dataset uses a variety of image creation methods, but it may still lack in certain real-world details, such as the stages at which bananas ripen or other factors that impact their quality. It’s essential to be aware of these limitations when analysing the results and applying the information in larger contexts.

## Ethics Statement

The study follows ethical guidelines for collecting and using data. We responsibly obtained all banana samples for this study from local sellers in Sirajganj, making sure that we did not harm the environment or the local people. The study was designed to be open and clear, showing exactly how the chemical treatment and picture collection were done. This study did not involve any animals or people, and it only looked at farm goods. The study follows local rules about food safety and chemical use, and it properly recognizes the effects of using calcium carbide. The results focus on improving food quality and safety and helping the public understand how toxic methods in farming affect food.

## CRediT Author Statement

**Elman Alam:** Data curation, Formal analysis, Software, Conceptualization, Methodology, Investigation, Writing – original draft, Visualization. **Md. Tarequl Islam:** Conceptualization, Formal analysis, Project administration, Supervision, Writing – review & editing, Methodology, Resources. **Ishrat Zahan Raka:** Formal analysis, Conceptualization, Project administration, Supervision, Writing – review & editing. **Onamika Sarkar Ritu:** Data curation, Formal analysis, Writing – original draft, Resources, Methodology, Software, Investigation. **Md Shakhawat Hossain:** Conceptualization, Formal analysis, Supervision, Project administration, Writing – review & editing, Software, Validation. **Md. Wahidur Rahman:** Conceptualization, Formal analysis, Supervision, Project administration, Writing – review & editing, Software, Validation. **Rahat Khan:** Conceptualization, Formal analysis, Supervision, Project administration, Writing – review & editing, Software, Validation.

## Data Availability

Mendeley DataRipen Banana Dataset: A Comprehensive Resource for Carbide Detection and Ripening Stage Analysis to Enhance Food Quality and Agricultural Efficiency (Original data).
